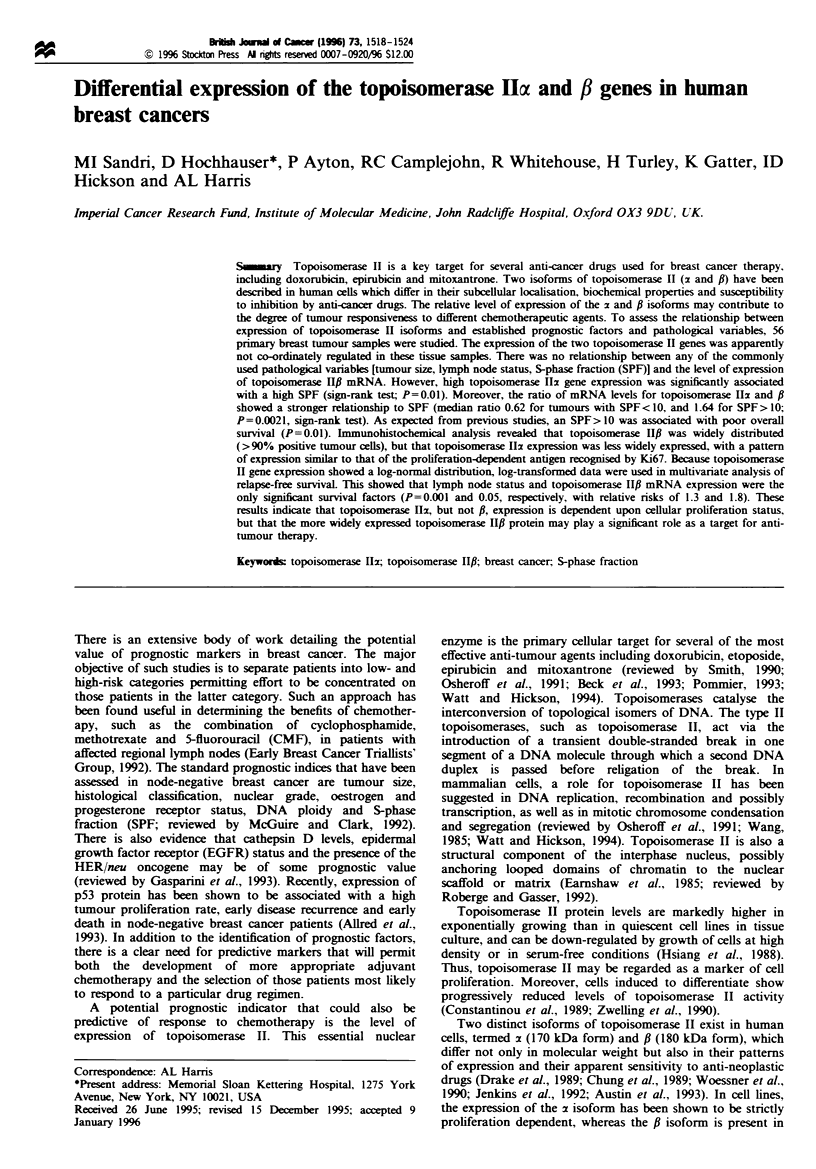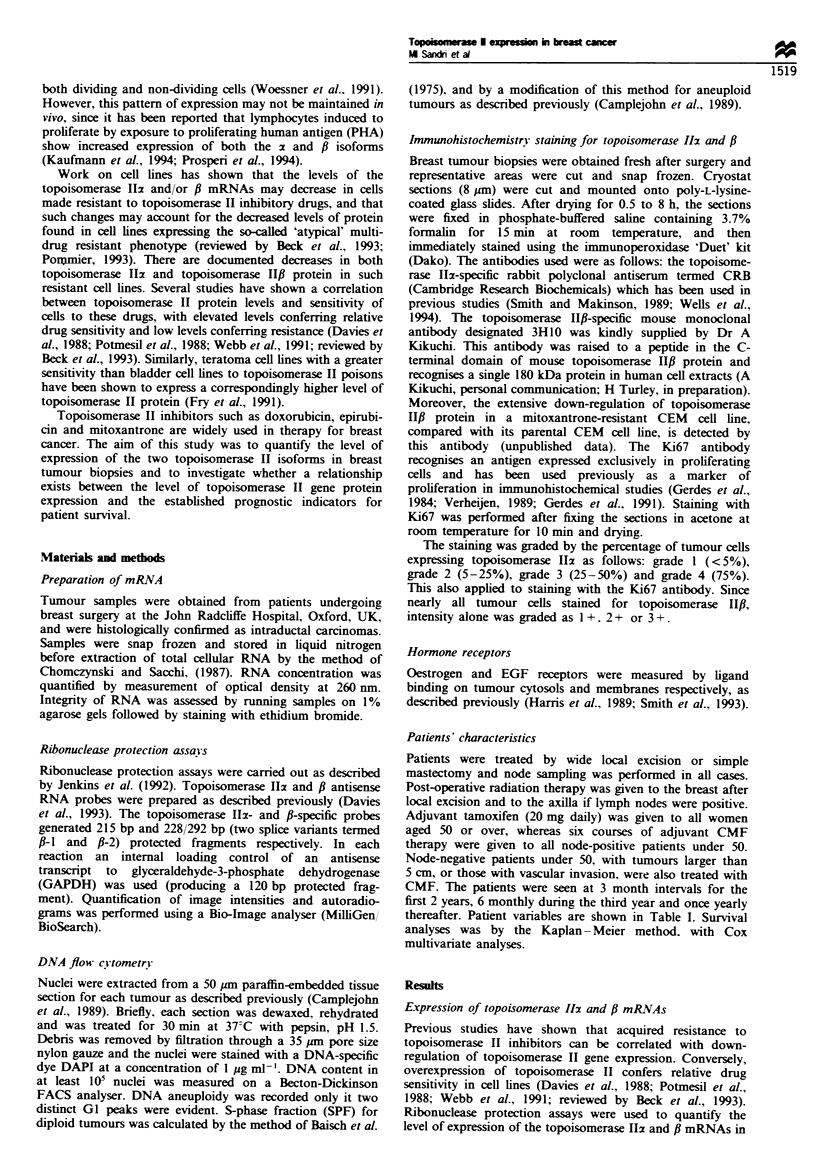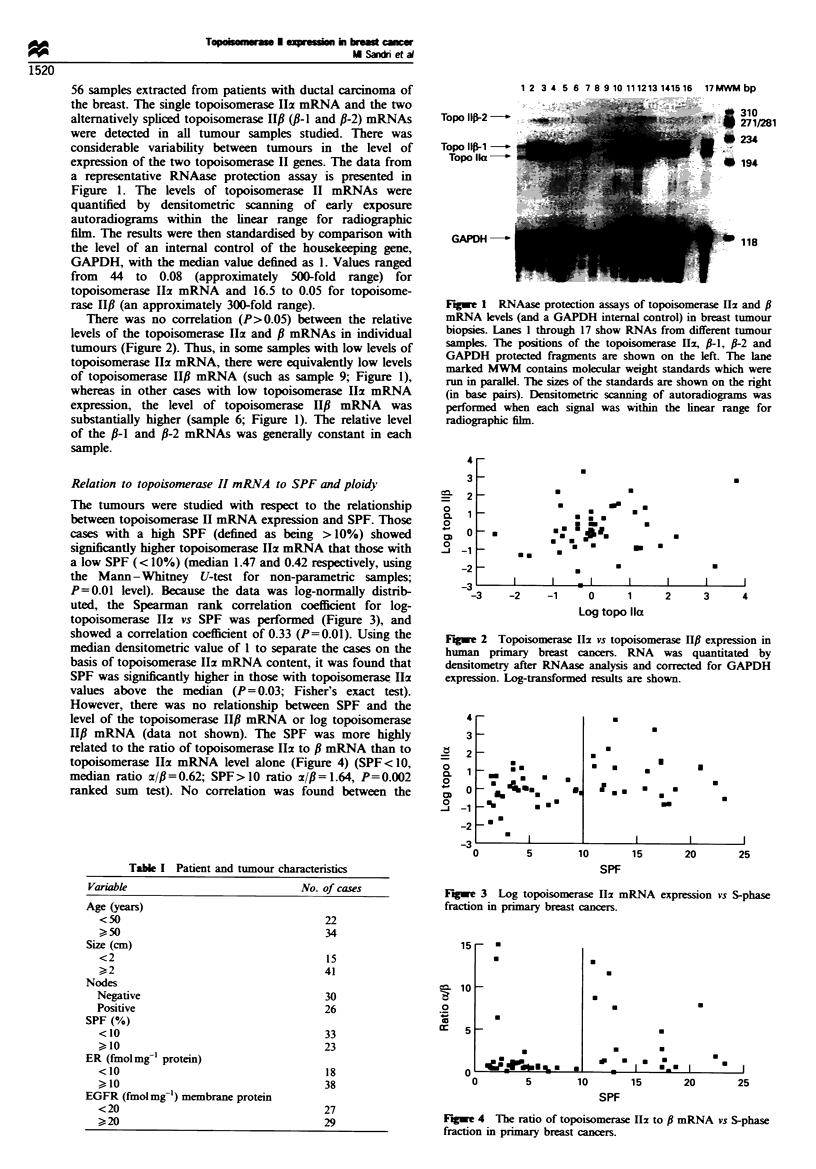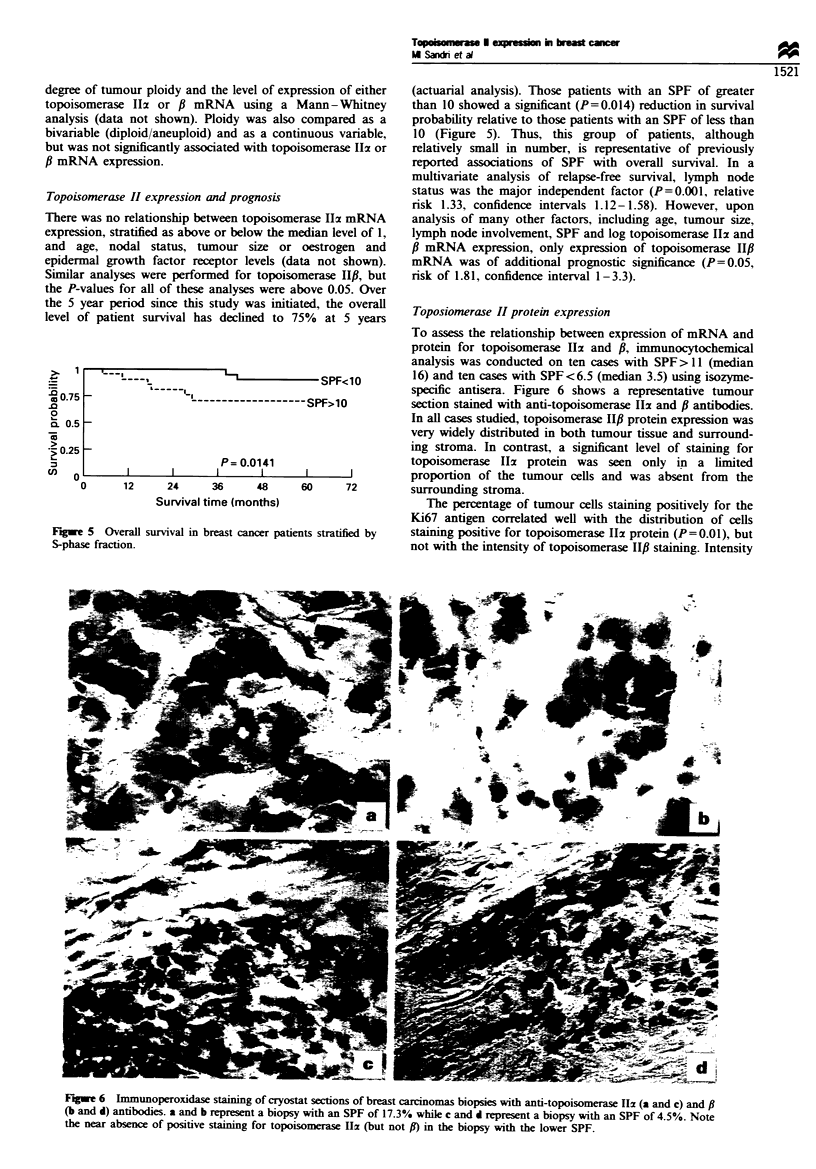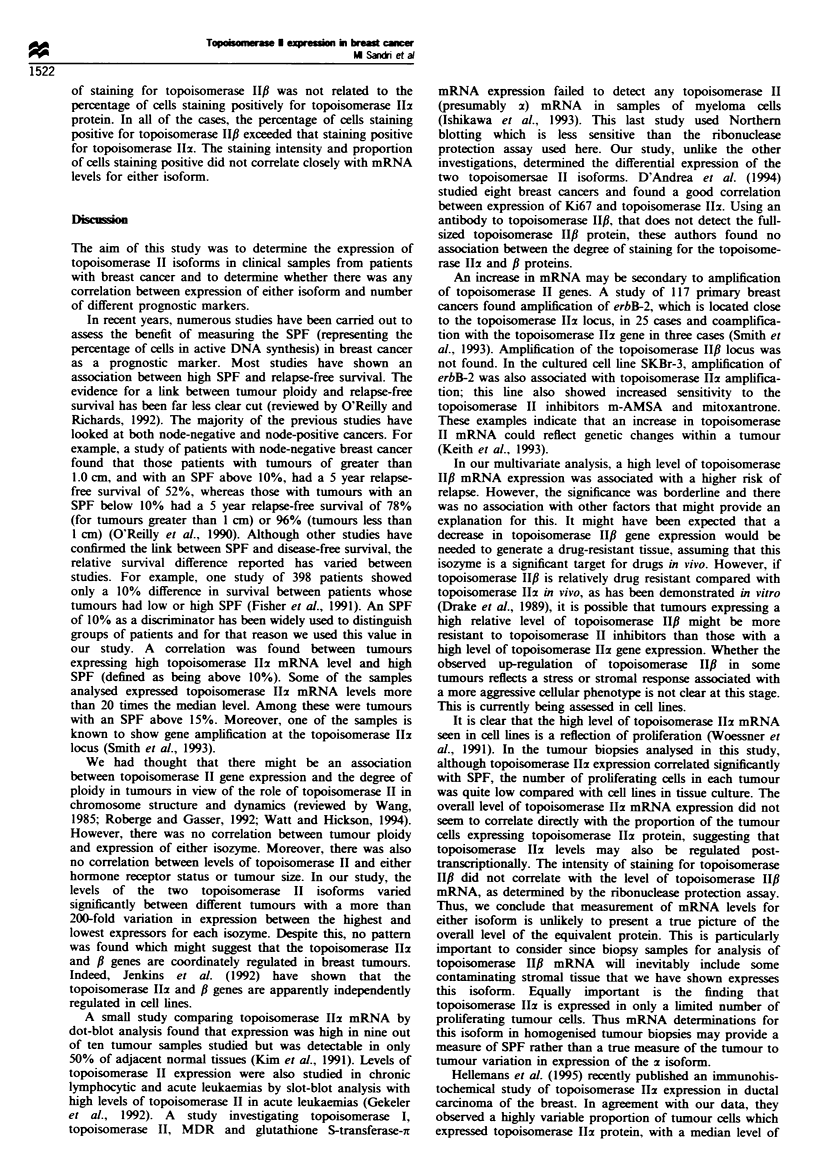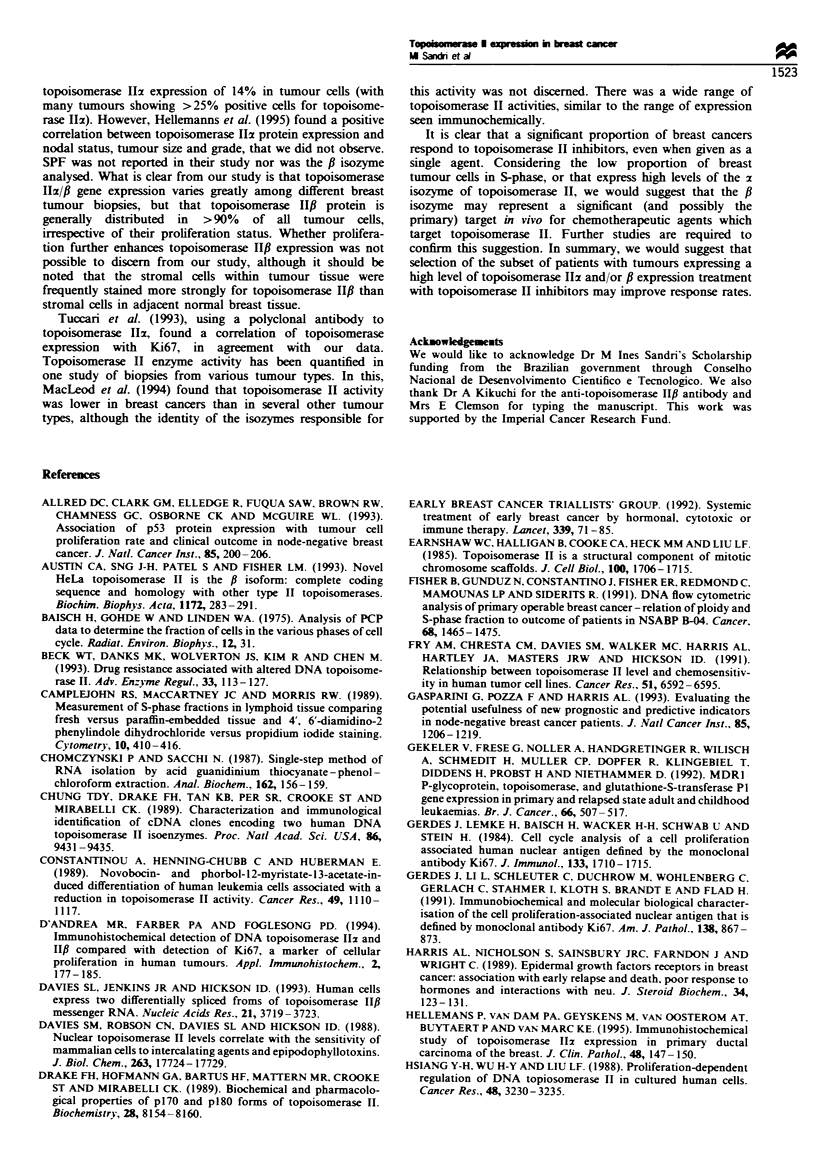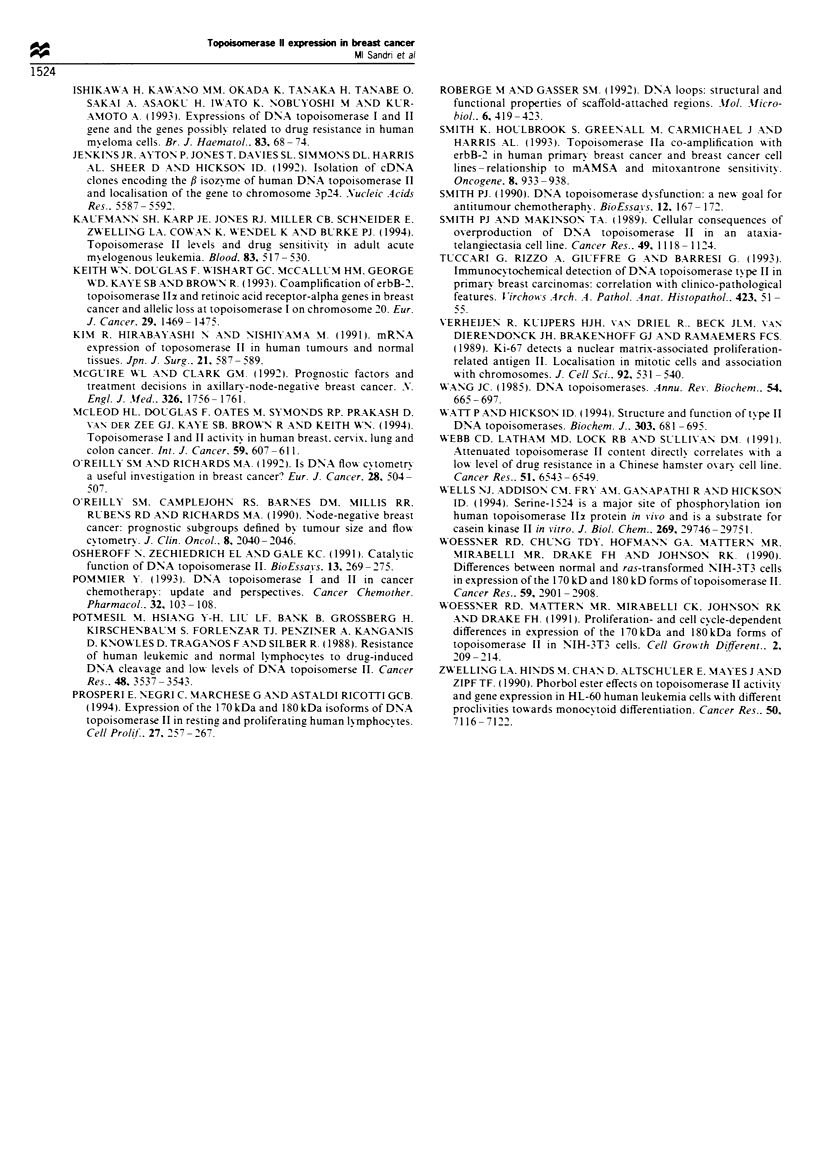# Differential expression of the topoisomerase II alpha and beta genes in human breast cancers.

**DOI:** 10.1038/bjc.1996.286

**Published:** 1996-06

**Authors:** M. I. Sandri, D. Hochhauser, P. Ayton, R. C. Camplejohn, R. Whitehouse, H. Turley, K. Gatter, I. D. Hickson, A. L. Harris

**Affiliations:** Imperial Cancer Research Fund, Institute of Molecular Medicine, John Radcliffe Hospital, Oxford, UK.

## Abstract

**Images:**


British Journal of Cancer (1996) 73, 1518–1524

© 1996 Stockton Press All rights reserved 0007-0920/96 $12.00

### Differential expression of the topoisomerase IIα and β genes in human breast cancers

MI Sandri, D Hochhauser*, P Ayton, RC Camplejohn, R Whitehouse, H Turley, K Gatter, ID Hickson and AL Harris

*Imperial Cancer Research Fund, Institute of Molecular Medicine, John Radcliffe Hospital, Oxford OX3 9DU, UK.*

**Summary** Topoisomerase II is a key target for several anti-cancer drugs used for breast cancer therapy, including doxorubicin, epirubicin and mitoxantrone. Two isoforms of topoisomerase II (α and β) have been described in human cells which differ in their subcellular localisation, biochemical properties and susceptibility to inhibition by anti-cancer drugs. The relative level of expression of the α and β isoforms may contribute to the degree of tumour responsiveness to different chemotherapeutic agents. To assess the relationship between expression of topoisomerase II isoforms and established prognostic factors and pathological variables, 56 primary breast tumour samples were studied. The expression of the two topoisomerase II genes was apparently not co-ordinately regulated in these tissue samples. There was no relationship between any of the commonly used pathological variables [tumour size, lymph node status, S-phase fraction (SPF)] and the level of expression of topoisomerase IIβ mRNA. However, high topoisomerase IIα gene expression was significantly associated with a high SPF (sign-rank test; $P=0.01$). Moreover, the ratio of mRNA levels for topoisomerase IIα and β showed a stronger relationship to SPF (median ratio 0.62 for tumours with SPF<10, and 1.64 for SPF>10; $P=0.0021$, sign-rank test). As expected from previous studies, an SPF>10 was associated with poor overall survival ($P=0.01$). Immunohistochemical analysis revealed that topoisomerase IIβ was widely distributed (>90% positive tumour cells), but that topoisomerase IIα expression was less widely expressed, with a pattern of expression similar to that of the proliferation-dependent antigen recognised by Ki67. Because topoisomerase II gene expression showed a log-normal distribution, log-transformed data were used in multivariate analysis of relapse-free survival. This showed that lymph node status and topoisomerase IIβ mRNA expression were the only significant survival factors ($P=0.001$ and 0.05, respectively, with relative risks of 1.3 and 1.8). These results indicate that topoisomerase IIα, but not β, expression is dependent upon cellular proliferation status, but that the more widely expressed topoisomerase IIβ protein may play a significant role as a target for anti-tumour therapy.

**Keywords:** topoisomerase IIα; topoisomerase IIβ; breast cancer; S-phase fraction

There is an extensive body of work detailing the potential value of prognostic markers in breast cancer. The major objective of such studies is to separate patients into low- and high-risk categories permitting effort to be concentrated on those patients in the latter category. Such an approach has been found useful in determining the benefits of chemotherapy, such as the combination of cyclophosphamide, methotrexate and 5-fluorouracil (CMF), in patients with affected regional lymph nodes (Early Breast Cancer Triallists' Group, 1992). The standard prognostic indices that have been assessed in node-negative breast cancer are tumour size, histological classification, nuclear grade, oestrogen and progesterone receptor status, DNA ploidy and S-phase fraction (SPF; reviewed by McGuire and Clark, 1992). There is also evidence that cathepsin D levels, epidermal growth factor receptor (EGFR) status and the presence of the HER/*neu* oncogene may be of some prognostic value (reviewed by Gasparini *et al.*, 1993). Recently, expression of p53 protein has been shown to be associated with a high tumour proliferation rate, early disease recurrence and early death in node-negative breast cancer patients (Allred *et al.*, 1993). In addition to the identification of prognostic factors, there is a clear need for predictive markers that will permit both the development of more appropriate adjuvant chemotherapy and the selection of those patients most likely to respond to a particular drug regimen.

A potential prognostic indicator that could also be predictive of response to chemotherapy is the level of expression of topoisomerase II. This essential nuclear enzyme is the primary cellular target for several of the most effective anti-tumour agents including doxorubicin, etoposide, epirubicin and mitoxantrone (reviewed by Smith, 1990; Osheroff *et al.*, 1991; Beck *et al.*, 1993; Pommier, 1993; Watt and Hickson, 1994). Topoisomerases catalyse the interconversion of topological isomers of DNA. The type II topoisomerases, such as topoisomerase II, act via the introduction of a transient double-stranded break in one segment of a DNA molecule through which a second DNA duplex is passed before religation of the break. In mammalian cells, a role for topoisomerase II has been suggested in DNA replication, recombination and possibly transcription, as well as in mitotic chromosome condensation and segregation (reviewed by Osheroff *et al.*, 1991; Wang, 1985; Watt and Hickson, 1994). Topoisomerase II is also a structural component of the interphase nucleus, possibly anchoring looped domains of chromatin to the nuclear scaffold or matrix (Earnshaw *et al.*, 1985; reviewed by Roberge and Gasser, 1992).

Topoisomerase II protein levels are markedly higher in exponentially growing than in quiescent cell lines in tissue culture, and can be down-regulated by growth of cells at high density or in serum-free conditions (Hsiang *et al.*, 1988). Thus, topoisomerase II may be regarded as a marker of cell proliferation. Moreover, cells induced to differentiate show progressively reduced levels of topoisomerase II activity (Constantinou *et al.*, 1989; Zwelling *et al.*, 1990).

Two distinct isoforms of topoisomerase II exist in human cells, termed α (170 kDa form) and β (180 kDa form), which differ not only in molecular weight but also in their patterns of expression and their apparent sensitivity to anti-neoplastic drugs (Drake *et al.*, 1989; Chung *et al.*, 1989; Woessner *et al.*, 1990; Jenkins *et al.*, 1992; Austin *et al.*, 1993). In cell lines, the expression of the α isoform has been shown to be strictly proliferation dependent, whereas the β isoform is present in

Correspondence: AL Harris
*Present address: Memorial Sloan Kettering Hospital, 1275 York Avenue, New York, NY 10021, USA
Received 26 June 1995; revised 15 December 1995; accepted 9 January 1996

both dividing and non-dividing cells (Woessner *et al.*, 1991). However, this pattern of expression may not be maintained *in vivo*, since it has been reported that lymphocytes induced to proliferate by exposure to proliferating human antigen (PHA) show increased expression of both the α and β isoforms (Kaufmann *et al.*, 1994; Prosperi *et al.*, 1994).

Work on cell lines has shown that the levels of the topoisomerase IIα and/or β mRNAs may decrease in cells made resistant to topoisomerase II inhibitory drugs, and that such changes may account for the decreased levels of protein found in cell lines expressing the so-called 'atypical' multidrug resistant phenotype (reviewed by Beck *et al.*, 1993; Pommier, 1993). There are documented decreases in both topoisomerase IIα and topoisomerase IIβ protein in such resistant cell lines. Several studies have shown a correlation between topoisomerase II protein levels and sensitivity of cells to these drugs, with elevated levels conferring relative drug sensitivity and low levels conferring resistance (Davies *et al.*, 1988; Potmesil *et al.*, 1988; Webb *et al.*, 1991; reviewed by Beck *et al.*, 1993). Similarly, teratoma cell lines with a greater sensitivity than bladder cell lines to topoisomerase II poisons have been shown to express a correspondingly higher level of topoisomerase II protein (Fry *et al.*, 1991).

Topoisomerase II inhibitors such as doxorubicin, epirubicin and mitoxantrone are widely used in therapy for breast cancer. The aim of this study was to quantify the level of expression of the two topoisomerase II isoforms in breast tumour biopsies and to investigate whether a relationship exists between the level of topoisomerase II gene protein expression and the established prognostic indicators for patient survival.

### Materials and methods

### *Preparation of mRNA*

Tumour samples were obtained from patients undergoing breast surgery at the John Radcliffe Hospital, Oxford, UK, and were histologically confirmed as intraductal carcinomas. Samples were snap frozen and stored in liquid nitrogen before extraction of total cellular RNA by the method of Chomczynski and Sacchi, (1987). RNA concentration was quantified by measurement of optical density at 260 nm. Integrity of RNA was assessed by running samples on 1% agarose gels followed by staining with ethidium bromide.

### *Ribonuclease protection assays*

Ribonuclease protection assays were carried out as described by Jenkins *et al.* (1992). Topoisomerase IIα and β antisense RNA probes were prepared as described previously (Davies *et al.*, 1993). The topoisomerase IIα- and β-specific probes generated 215 bp and 228/292 bp (two splice variants termed β-1 and β-2) protected fragments respectively. In each reaction an internal loading control of an antisense transcript to glyceraldehyde-3-phosphate dehydrogenase (GAPDH) was used (producing a 120 bp protected fragment). Quantification of image intensities and autoradiograms was performed using a Bio-Image analyser (MilliGen/BioSearch).

### *DNA flow cytometry*

Nuclei were extracted from a 50 μm paraffin-embedded tissue section for each tumour as described previously (Camplejohn *et al.*, 1989). Briefly, each section was dewaxed, rehydrated and was treated for 30 min at 37°C with pepsin, pH 1.5. Debris was removed by filtration through a 35 μm pore size nylon gauze and the nuclei were stained with a DNA-specific dye DAPI at a concentration of 1 μg $ml^{-1}$. DNA content in at least $10^5$ nuclei was measured on a Becton-Dickinson FACS analyser. DNA aneuploidy was recorded only it two distinct G1 peaks were evident. S-phase fraction (SPF) for diploid tumours was calculated by the method of Baisch *et al.* (1975), and by a modification of this method for aneuploid tumours as described previously (Camplejohn *et al.*, 1989).

### *Immunohistochemistry staining for topoisomerase IIα and β*

Breast tumour biopsies were obtained fresh after surgery and representative areas were cut and snap frozen. Cryostat sections (8 μm) were cut and mounted onto poly-L-lysine-coated glass slides. After drying for 0.5 to 8 h, the sections were fixed in phosphate-buffered saline containing 3.7% formalin for 15 min at room temperature, and then immediately stained using the immunoperoxidase 'Duet' kit (Dako). The antibodies used were as follows: the topoisomerase IIα-specific rabbit polyclonal antiserum termed CRB (Cambridge Research Biochemicals) which has been used in previous studies (Smith and Makinson, 1989; Wells *et al.*, 1994). The topoisomerase IIβ-specific mouse monoclonal antibody designated 3H10 was kindly supplied by Dr A Kikuchi. This antibody was raised to a peptide in the C-terminal domain of mouse topoisomerase IIβ protein and recognises a single 180 kDa protein in human cell extracts (A Kikuchi, personal communication; H Turley, in preparation). Moreover, the extensive down-regulation of topoisomerase IIβ protein in a mitoxantrone-resistant CEM cell line, compared with its parental CEM cell line, is detected by this antibody (unpublished data). The Ki67 antibody recognises an antigen expressed exclusively in proliferating cells and has been used previously as a marker of proliferation in immunohistochemical studies (Gerdes *et al.*, 1984; Verheijen, 1989; Gerdes *et al.*, 1991). Staining with Ki67 was performed after fixing the sections in acetone at room temperature for 10 min and drying.

The staining was graded by the percentage of tumour cells expressing topoisomerase IIα as follows: grade 1 (<5%), grade 2 (5–25%), grade 3 (25–50%) and grade 4 (75%). This also applied to staining with the Ki67 antibody. Since nearly all tumour cells stained for topoisomerase IIβ, intensity alone was graded as 1+, 2+ or 3+.

### *Hormone receptors*

Oestrogen and EGF receptors were measured by ligand binding on tumour cytosols and membranes respectively, as described previously (Harris *et al.*, 1989; Smith *et al.*, 1993).

### *Patients' characteristics*

Patients were treated by wide local excision or simple mastectomy and node sampling was performed in all cases. Post-operative radiation therapy was given to the breast after local excision and to the axilla if lymph nodes were positive. Adjuvant tamoxifen (20 mg daily) was given to all women aged 50 or over, whereas six courses of adjuvant CMF therapy were given to all node-positive patients under 50. Node-negative patients under 50, with tumours larger than 5 cm, or those with vascular invasion, were also treated with CMF. The patients were seen at 3 month intervals for the first 2 years, 6 monthly during the third year and once yearly thereafter. Patient variables are shown in Table I. Survival analyses was by the Kaplan–Meier method, with Cox multivariate analyses.

### Results

### *Expression of topoisomerase IIα and β mRNAs*

Previous studies have shown that acquired resistance to topoisomerase II inhibitors can be correlated with down-regulation of topoisomerase II gene expression. Conversely, overexpression of topoisomerase II confers relative drug sensitivity in cell lines (Davies *et al.*, 1988; Potmesil *et al.*, 1988; Webb *et al.*, 1991; reviewed by Beck *et al.*, 1993). Ribonuclease protection assays were used to quantify the level of expression of the topoisomerase IIα and β mRNAs in

56 samples extracted from patients with ductal carcinoma of the breast. The single topoisomerase IIα mRNA and the two alternatively spliced topoisomerase IIβ (β-1 and β-2) mRNAs were detected in all tumour samples studied. There was considerable variability between tumours in the level of expression of the two topoisomerase II genes. The data from a representative RNAase protection assay is presented in Figure 1. The levels of topoisomerase II mRNAs were quantified by densitometric scanning of early exposure autoradiograms within the linear range for radiographic film. The results were then standardised by comparison with the level of an internal control of the housekeeping gene, GAPDH, with the median value defined as 1. Values ranged from 44 to 0.08 (approximately 500-fold range) for topoisomerase IIα mRNA and 16.5 to 0.05 for topoisomerase IIβ (an approximately 300-fold range).

There was no correlation ($P>0.05$) between the relative levels of the topoisomerase IIα and β mRNAs in individual tumours (Figure 2). Thus, in some samples with low levels of topoisomerase IIα mRNA, there were equivalently low levels of topoisomerase IIβ mRNA (such as sample 9; Figure 1), whereas in other cases with low topoisomerase IIα mRNA expression, the level of topoisomerase IIβ mRNA was substantially higher (sample 6; Figure 1). The relative level of the β-1 and β-2 mRNAs was generally constant in each sample.

*Relation to topoisomerase II mRNA to SPF and ploidy*

The tumours were studied with respect to the relationship between topoisomerase II mRNA expression and SPF. Those cases with a high SPF (defined as being >10%) showed significantly higher topoisomerase IIα mRNA that those with a low SPF (<10%) (median 1.47 and 0.42 respectively, using the Mann–Whitney *U*-test for non-parametric samples; $P=0.01$ level). Because the data was log-normally distributed, the Spearman rank correlation coefficient for log-topoisomerase IIα *vs* SPF was performed (Figure 3), and showed a correlation coefficient of 0.33 ($P=0.01$). Using the median densitometric value of 1 to separate the cases on the basis of topoisomerase IIα mRNA content, it was found that SPF was significantly higher in those with topoisomerase IIα values above the median ($P=0.03$; Fisher's exact test). However, there was no relationship between SPF and the level of the topoisomerase IIβ mRNA or log topoisomerase IIβ mRNA (data not shown). The SPF was more highly related to the ratio of topoisomerase IIα to β mRNA than to topoisomerase IIα mRNA level alone (Figure 4) (SPF<10, median ratio $\alpha/\beta=0.62$; SPF>10 ratio $\alpha/\beta=1.64$, $P=0.002$ ranked sum test). No correlation was found between the

**Table I** Patient and tumour characteristics

| *Variable* | *No. of cases* |
|---|---|
| Age (years) | |
| <50 | 22 |
| ⩾50 | 34 |
| Size (cm) | |
| <2 | 15 |
| ⩾2 | 41 |
| Nodes | |
| Negative | 30 |
| Positive | 26 |
| SPF (%) | |
| <10 | 33 |
| ⩾10 | 23 |
| ER (fmol mg$^{-1}$ protein) | |
| <10 | 18 |
| ⩾10 | 38 |
| EGFR (fmol mg$^{-1}$) membrane protein | |
| <20 | 27 |
| ⩾20 | 29 |

**Figure 1** RNAase protection assays of topoisomerase IIα and β mRNA levels (and a GAPDH internal control) in breast tumour biopsies. Lanes 1 through 17 show RNAs from different tumour samples. The positions of the topoisomerase IIα, β-1, β-2 and GAPDH protected fragments are shown on the left. The lane marked MWM contains molecular weight standards which were run in parallel. The sizes of the standards are shown on the right (in base pairs). Densitometric scanning of autoradiograms was performed when each signal was within the linear range for radiographic film.

**Figure 2** Topoisomerase IIα *vs* topoisomerase IIβ expression in human primary breast cancers. RNA was quantitated by densitometry after RNAase analysis and corrected for GAPDH expression. Log-transformed results are shown.

**Figure 3** Log topoisomerase IIα mRNA expression *vs* S-phase fraction in primary breast cancers.

**Figure 4** The ratio of topoisomerase IIα to β mRNA *vs* S-phase fraction in primary breast cancers.

degree of tumour ploidy and the level of expression of either topoisomerase IIα or β mRNA using a Mann–Whitney analysis (data not shown). Ploidy was also compared as a bivariable (diploid/aneuploid) and as a continuous variable, but was not significantly associated with topoisomerase IIα or β mRNA expression.

### *Topoisomerase II expression and prognosis*

There was no relationship between topoisomerase IIα mRNA expression, stratified as above or below the median level of 1, and age, nodal status, tumour size or oestrogen and epidermal growth factor receptor levels (data not shown). Similar analyses were performed for topoisomerase IIβ, but the $P$-values for all of these analyses were above 0.05. Over the 5 year period since this study was initiated, the overall level of patient survival has declined to 75% at 5 years

**Figure 5** Overall survival in breast cancer patients stratified by S-phase fraction.

(actuarial analysis). Those patients with an SPF of greater than 10 showed a significant ($P$=0.014) reduction in survival probability relative to those patients with an SPF of less than 10 (Figure 5). Thus, this group of patients, although relatively small in number, is representative of previously reported associations of SPF with overall survival. In a multivariate analysis of relapse-free survival, lymph node status was the major independent factor ($P$=0.001, relative risk 1.33, confidence intervals 1.12–1.58). However, upon analysis of many other factors, including age, tumour size, lymph node involvement, SPF and log topoisomerase IIα and β mRNA expression, only expression of topoisomerase IIβ mRNA was of additional prognostic significance ($P$=0.05, risk of 1.81, confidence interval 1–3.3).

### *Toposiomerase II protein expression*

To assess the relationship between expression of mRNA and protein for topoisomerase IIα and β, immunocytochemical analysis was conducted on ten cases with SPF>11 (median 16) and ten cases with SPF<6.5 (median 3.5) using isozyme-specific antisera. Figure 6 shows a representative tumour section stained with anti-topoisomerase IIα and β antibodies. In all cases studied, topoisomerase IIβ protein expression was very widely distributed in both tumour tissue and surrounding stroma. In contrast, a significant level of staining for topoisomerase IIα protein was seen only in a limited proportion of the tumour cells and was absent from the surrounding stroma.

The percentage of tumour cells staining positively for the Ki67 antigen correlated well with the distribution of cells staining positive for topoisomerase IIα protein ($P$=0.01), but not with the intensity of topoisomerase IIβ staining. Intensity

**Figure 6** Immunoperoxidase staining of cryostat sections of breast carcinomas biopsies with anti-topoisomerase IIα (**a** and **c**) and β (**b** and **d**) antibodies. **a** and **b** represent a biopsy with an SPF of 17.3% while **c** and **d** represent a biopsy with an SPF of 4.5%. Note the near absence of positive staining for topoisomerase IIα (but not β) in the biopsy with the lower SPF.

of staining for topoisomerase II$\beta$ was not related to the percentage of cells staining positively for topoisomerase II$\alpha$ protein. In all of the cases, the percentage of cells staining positive for topoisomerase II$\beta$ exceeded that staining positive for topoisomerase II$\alpha$. The staining intensity and proportion of cells staining positive did not correlate closely with mRNA levels for either isoform.

### Discussion

The aim of this study was to determine the expression of topoisomerase II isoforms in clinical samples from patients with breast cancer and to determine whether there was any correlation between expression of either isoform and number of different prognostic markers.

In recent years, numerous studies have been carried out to assess the benefit of measuring the SPF (representing the percentage of cells in active DNA synthesis) in breast cancer as a prognostic marker. Most studies have shown an association between high SPF and relapse-free survival. The evidence for a link between tumour ploidy and relapse-free survival has been far less clear cut (reviewed by O'Reilly and Richards, 1992). The majority of the previous studies have looked at both node-negative and node-positive cancers. For example, a study of patients with node-negative breast cancer found that those patients with tumours of greater than 1.0 cm, and with an SPF above 10%, had a 5 year relapse-free survival of 52%, whereas those with tumours with an SPF below 10% had a 5 year relapse-free survival of 78% (for tumours greater than 1 cm) or 96% (tumours less than 1 cm) (O'Reilly *et al*., 1990). Although other studies have confirmed the link between SPF and disease-free survival, the relative survival difference reported has varied between studies. For example, one study of 398 patients showed only a 10% difference in survival between patients whose tumours had low or high SPF (Fisher *et al*., 1991). An SPF of 10% as a discriminator has been widely used to distinguish groups of patients and for that reason we used this value in our study. A correlation was found between tumours expressing high topoisomerase II$\alpha$ mRNA level and high SPF (defined as being above 10%). Some of the samples analysed expressed topoisomerase II$\alpha$ mRNA levels more than 20 times the median level. Among these were tumours with an SPF above 15%. Moreover, one of the samples is known to show gene amplification at the topoisomerase II$\alpha$ locus (Smith *et al*., 1993).

We had thought that there might be an association between topoisomerase II gene expression and the degree of ploidy in tumours in view of the role of topoisomerase II in chromosome structure and dynamics (reviewed by Wang, 1985; Roberge and Gasser, 1992; Watt and Hickson, 1994). However, there was no correlation between tumour ploidy and expression of either isozyme. Moreover, there was also no correlation between levels of topoisomerase II and either hormone receptor status or tumour size. In our study, the levels of the two topoisomerase II isoforms varied significantly between different tumours with a more than 200-fold variation in expression between the highest and lowest expressors for each isozyme. Despite this, no pattern was found which might suggest that the topoisomerase II$\alpha$ and $\beta$ genes are coordinately regulated in breast tumours. Indeed, Jenkins *et al*. (1992) have shown that the topoisomerase II$\alpha$ and $\beta$ genes are apparently independently regulated in cell lines.

A small study comparing topoisomerase II$\alpha$ mRNA by dot-blot analysis found that expression was high in nine out of ten tumour samples studied but was detectable in only 50% of adjacent normal tissues (Kim *et al*., 1991). Levels of topoisomerase II expression were also studied in chronic lymphocytic and acute leukaemias by slot-blot analysis with high levels of topoisomerase II in acute leukaemias (Gekeler *et al*., 1992). A study investigating topoisomerase I, topoisomerase II, MDR and glutathione S-transferase-$\pi$ mRNA expression failed to detect any topoisomerase II (presumably $\alpha$) mRNA in samples of myeloma cells (Ishikawa *et al*., 1993). This last study used Northern blotting which is less sensitive than the ribonuclease protection assay used here. Our study, unlike the other investigations, determined the differential expression of the two topoisomersae II isoforms. D'Andrea *et al*. (1994) studied eight breast cancers and found a good correlation between expression of Ki67 and topoisomerase II$\alpha$. Using an antibody to topoisomerase II$\beta$, that does not detect the full-sized topoisomerase II$\beta$ protein, these authors found no association between the degree of staining for the topoisomerase II$\alpha$ and $\beta$ proteins.

An increase in mRNA may be secondary to amplification of topoisomerase II genes. A study of 117 primary breast cancers found amplification of *erb*B-2, which is located close to the topoisomerase II$\alpha$ locus, in 25 cases and coamplification with the topoisomerase II$\alpha$ gene in three cases (Smith *et al*., 1993). Amplification of the topoisomerase II$\beta$ locus was not found. In the cultured cell line SKBr-3, amplification of *erb*B-2 was also associated with topoisomerase II$\alpha$ amplification; this line also showed increased sensitivity to the topoisomerase II inhibitors m-AMSA and mitoxantrone. These examples indicate that an increase in topoisomerase II mRNA could reflect genetic changes within a tumour (Keith *et al*., 1993).

In our multivariate analysis, a high level of topoisomerase II$\beta$ mRNA expression was associated with a higher risk of relapse. However, the significance was borderline and there was no association with other factors that might provide an explanation for this. It might have been expected that a decrease in topoisomerase II$\beta$ gene expression would be needed to generate a drug-resistant tissue, assuming that this isozyme is a significant target for drugs *in vivo*. However, if topoisomerase II$\beta$ is relatively drug resistant compared with topoisomerase II$\alpha$ *in vivo*, as has been demonstrated *in vitro* (Drake *et al*., 1989), it is possible that tumours expressing a high relative level of topoisomerase II$\beta$ might be more resistant to topoisomerase II inhibitors than those with a high level of topoisomerase II$\alpha$ gene expression. Whether the observed up-regulation of topoisomerase II$\beta$ in some tumours reflects a stress or stromal response associated with a more aggressive cellular phenotype is not clear at this stage. This is currently being assessed in cell lines.

It is clear that the high level of topoisomerase II$\alpha$ mRNA seen in cell lines is a reflection of proliferation (Woessner *et al*., 1991). In the tumour biopsies analysed in this study, although topoisomerase II$\alpha$ expression correlated significantly with SPF, the number of proliferating cells in each tumour was quite low compared with cell lines in tissue culture. The overall level of topoisomerase II$\alpha$ mRNA expression did not seem to correlate directly with the proportion of the tumour cells expressing topoisomerase II$\alpha$ protein, suggesting that topoisomerase II$\alpha$ levels may also be regulated post-transcriptionally. The intensity of staining for topoisomerase II$\beta$ did not correlate with the level of topoisomerase II$\beta$ mRNA, as determined by the ribonuclease protection assay. Thus, we conclude that measurement of mRNA levels for either isoform is unlikely to present a true picture of the overall level of the equivalent protein. This is particularly important to consider since biopsy samples for analysis of topoisomerase II$\beta$ mRNA will inevitably include some contaminating stromal tissue that we have shown expresses this isoform. Equally important is the finding that topoisomerase II$\alpha$ is expressed in only a limited number of proliferating tumour cells. Thus mRNA determinations for this isoform in homogenised tumour biopsies may provide a measure of SPF rather than a true measure of the tumour to tumour variation in expression of the $\alpha$ isoform.

Hellemans *et al*. (1995) recently published an immunohistochemical study of topoisomerase II$\alpha$ expression in ductal carcinoma of the breast. In agreement with our data, they observed a highly variable proportion of tumour cells which expressed topoisomerase II$\alpha$ protein, with a median level of topoisomerase IIα expression of 14% in tumour cells (with many tumours showing >25% positive cells for topoisomerase IIα). However, Hellemanns *et al.* (1995) found a positive correlation between topoisomerase IIα protein expression and nodal status, tumour size and grade, that we did not observe. SPF was not reported in their study nor was the β isozyme analysed. What is clear from our study is that topoisomerase IIα/β gene expression varies greatly among different breast tumour biopsies, but that topoisomerase IIβ protein is generally distributed in >90% of all tumour cells, irrespective of their proliferation status. Whether proliferation further enhances topoisomerase IIβ expression was not possible to discern from our study, although it should be noted that the stromal cells within tumour tissue were frequently stained more strongly for topoisomerase IIβ than stromal cells in adjacent normal breast tissue.

Tuccari *et al.* (1993), using a polyclonal antibody to topoisomerase IIα, found a correlation of topoisomerase expression with Ki67, in agreement with our data. Topoisomerase II enzyme activity has been quantified in one study of biopsies from various tumour types. In this, MacLeod *et al.* (1994) found that topoisomerase II activity was lower in breast cancers than in several other tumour types, although the identity of the isozymes responsible for this activity was not discerned. There was a wide range of topoisomerase II activities, similar to the range of expression seen immunochemically.

It is clear that a significant proportion of breast cancers respond to topoisomerase II inhibitors, even when given as a single agent. Considering the low proportion of breast tumour cells in S-phase, or that express high levels of the α isozyme of topoisomerase II, we would suggest that the β isozyme may represent a significant (and possibly the primary) target *in vivo* for chemotherapeutic agents which target topoisomerase II. Further studies are required to confirm this suggestion. In summary, we would suggest that selection of the subset of patients with tumours expressing a high level of topoisomerase IIα and/or β expression treatment with topoisomerase II inhibitors may improve response rates.

**Acknowledgements**

We would like to acknowledge Dr M Ines Sandri's Scholarship funding from the Brazilian government through Conselho Nacional de Desenvolvimento Cientifico e Tecnologico. We also thank Dr A Kikuchi for the anti-topoisomerase IIβ antibody and Mrs E Clemson for typing the manuscript. This work was supported by the Imperial Cancer Research Fund.

### References

ALLRED DC, CLARK GM, ELLEDGE R, FUQUA SAW, BROWN RW, CHAMNESS GC, OSBORNE CK AND MCGUIRE WL. (1993). Association of p53 protein expression with tumour cell proliferation rate and clinical outcome in node-negative breast cancer. *J. Natl. Cancer Inst.*, **85**, 200–206.

AUSTIN CA, SNG J-H, PATEL S AND FISHER LM. (1993). Novel HeLa topoisomerase II is the β isoform: complete coding sequence and homology with other type II topoisomerases. *Biochim. Biophys. Acta*, **1172**, 283–291.

BAISCH H, GOHDE W AND LINDEN WA. (1975). Analysis of PCP data to determine the fraction of cells in the various phases of cell cycle. *Radiat. Environ. Biophys.*, **12**, 31.

BECK WT, DANKS MK, WOLVERTON JS, KIM R AND CHEN M. (1993). Drug resistance associated with altered DNA topoisomerase II. *Adv. Enzyme Regul.*, **33**, 113–127.

CAMPLEJOHN RS, MACCARTNEY JC AND MORRIS RW. (1989). Measurement of S-phase fractions in lymphoid tissue comparing fresh versus paraffin-embedded tissue and 4′, 6′-diamidino-2 phenylindole dihydrochloride versus propidium iodide staining. *Cytometry*, **10**, 410–416.

CHOMCZYNSKI P AND SACCHI N. (1987). Single-step method of RNA isolation by acid guanidinium thiocyanate–phenol–chloroform extraction. *Anal. Biochem.*, **162**, 156–159.

CHUNG TDY, DRAKE FH, TAN KB, PER SR, CROOKE ST AND MIRABELLI CK. (1989). Characterization and immunological identification of cDNA clones encoding two human DNA topoisomerase II isoenzymes. *Proc. Natl Acad. Sci. USA*, **86**, 9431–9435.

CONSTANTINOU A, HENNING-CHUBB C AND HUBERMAN E. (1989). Novobocin- and phorbol-12-myristate-13-acetate-induced differentiation of human leukemia cells associated with a reduction in topoisomerase II activity. *Cancer Res.*, **49**, 1110–1117.

D'ANDREA MR, FARBER PA AND FOGLESONG PD. (1994). Immunohistochemical detection of DNA topoisomerase IIα and IIβ compared with detection of Ki67, a marker of cellular proliferation in human tumours. *Appl. Immunohistochem.*, **2**, 177–185.

DAVIES SL, JENKINS JR AND HICKSON ID. (1993). Human cells express two differentially spliced froms of topoisomerase IIβ messenger RNA. *Nucleic Acids Res.*, **21**, 3719–3723.

DAVIES SM, ROBSON CN, DAVIES SL AND HICKSON ID. (1988). Nuclear topoisomerase II levels correlate with the sensitivity of mammalian cells to intercalating agents and epipodophyllotoxins. *J. Biol. Chem.*, **263**, 17724–17729.

DRAKE FH, HOFMANN GA, BARTUS HF, MATTERN MR, CROOKE ST AND MIRABELLI CK. (1989). Biochemical and pharmacological properties of p170 and p180 forms of topoisomerase II. *Biochemistry*, **28**, 8154–8160.

EARLY BREAST CANCER TRIALLISTS' GROUP. (1992). Systemic treatment of early breast cancer by hormonal, cytotoxic or immune therapy. *Lancet*, **339**, 71–85.

EARNSHAW WC, HALLIGAN B, COOKE CA, HECK MM AND LIU LF. (1985). Topoisomerase II is a structural component of mitotic chromosome scaffolds. *J. Cell Biol.*, **100**, 1706–1715.

FISHER B, GUNDUZ N, CONSTANTINO J, FISHER ER, REDMOND C, MAMOUNAS LP AND SIDERITS R. (1991). DNA flow cytometric analysis of primary operable breast cancer – relation of ploidy and S-phase fraction to outcome of patients in NSABP B-04. *Cancer*, **68**, 1465–1475.

FRY AM, CHRESTA CM, DAVIES SM, WALKER MC, HARRIS AL, HARTLEY JA, MASTERS JRW AND HICKSON ID. (1991). Relationship between topoisomerase II level and chemosensitivity in human tumor cell lines. *Cancer Res.*, **51**, 6592–6595.

GASPARINI G, POZZA F AND HARRIS AL. (1993). Evaluating the potential usefulness of new prognostic and predictive indicators in node-negative breast cancer patients. *J. Natl Cancer Inst.*, **85**, 1206–1219.

GEKELER V, FRESE G, NOLLER A, HANDGRETINGER R, WILISCH A, SCHMEDIT H, MULLER CP, DOPFER R, KLINGEBIEL T, DIDDENS H, PROBST H AND NIETHAMMER D. (1992). MDR1/P-glycoprotein, topoisomerase, and glutathione-S-transferase P1 gene expression in primary and relapsed state adult and childhood leukaemias. *Br. J. Cancer.*, **66**, 507–517.

GERDES J, LEMKE H, BAISCH H, WACKER H-H, SCHWAB U AND STEIN H. (1984). Cell cycle analysis of a cell proliferation associated human nuclear antigen defined by the monoclonal antibody Ki67. *J. Immunol.*, **133**, 1710–1715.

GERDES J, LI L, SCHLEUTER C, DUCHROW M, WOHLENBERG C, GERLACH C, STAHMER I, KLOTH S, BRANDT E AND FLAD H. (1991). Immunobiochemical and molecular biological characterisation of the cell proliferation-associated nuclear antigen that is defined by monoclonal antibody Ki67. *Am. J. Pathol.*, **138**, 867–873.

HARRIS AL, NICHOLSON S, SAINSBURY JRC, FARNDON J AND WRIGHT C. (1989). Epidermal growth factors receptors in breast cancer: association with early relapse and death, poor response to hormones and interactions with neu. *J. Steroid Biochem.*, **34**, 123–131.

HELLEMANS P, VAN DAM PA, GEYSKENS M, VAN OOSTEROM AT, BUYTAERT P AND VAN MARC KE. (1995). Immunohistochemical study of topoisomerase IIα expression in primary ductal carcinoma of the breast. *J. Clin. Pathol.*, **48**, 147–150.

HSIANG Y-H, WU H-Y AND LIU LF. (1988). Proliferation-dependent regulation of DNA topiosomerase II in cultured human cells. *Cancer Res.*, **48**, 3230–3235.

ISHIKAWA H. KAWANO MM. OKADA K. TANAKA H. TANABE O. SAKAI A. ASAOKU H. IWATO K. NOBUYOSHI M AND KURAMOTO A. (1993). Expressions of DNA topoisomerase I and II gene and the genes possibly related to drug resistance in human myeloma cells. *Br. J. Haematol.*, **83**, 68–74.

JENKINS JR. AYTON P. JONES T. DAVIES SL. SIMMONS DL. HARRIS AL. SHEER D AND HICKSON ID. (1992). Isolation of cDNA clones encoding the $\beta$ isozyme of human DNA topoisomerase II and localisation of the gene to chromosome 3p24. *Nucleic Acids Res.*, 5587–5592.

KAUFMANN SH. KARP JE. JONES RJ. MILLER CB. SCHNEIDER E. ZWELLING LA. COWAN K. WENDEL K AND BURKE PJ. (1994). Topoisomerase II levels and drug sensitivity in adult acute myelogenous leukemia. *Blood*, **83**, 517–530.

KEITH WN. DOUGLAS F. WISHART GC. McCALLUM HM. GEORGE WD. KAYE SB AND BROWN R. (1993). Coamplification of erbB-2, topoisomerase II$\alpha$ and retinoic acid receptor-alpha genes in breast cancer and allelic loss at topoisomerase I on chromosome 20. *Eur. J. Cancer*, **29**, 1469–1475.

KIM R. HIRABAYASHI N AND NISHIYAMA M. (1991). mRNA expression of toposomerase II in human tumours and normal tissues. *Jpn. J. Surg.*, **21**, 587–589.

McGUIRE WL AND CLARK GM. (1992). Prognostic factors and treatment decisions in axillary-node-negative breast cancer. *N. Engl. J. Med.*, **326**, 1756–1761.

McLEOD HL. DOUGLAS F. OATES M. SYMONDS RP. PRAKASH D. VAN DER ZEE GJ. KAYE SB. BROWN R AND KEITH WN. (1994). Topoisomerase I and II activity in human breast, cervix, lung and colon cancer. *Int. J. Cancer*, **59**, 607–611.

O'REILLY SM AND RICHARDS MA. (1992). Is DNA flow cytometry a useful investigation in breast cancer? *Eur. J. Cancer*, **28**, 504–507.

O'REILLY SM. CAMPLEJOHN RS. BARNES DM. MILLIS RR. RUBENS RD AND RICHARDS MA. (1990). Node-negative breast cancer: prognostic subgroups defined by tumour size and flow cytometry. *J. Clin. Oncol.*, **8**, 2040–2046.

OSHEROFF N. ZECHIEDRICH EL AND GALE KC. (1991). Catalytic function of DNA topoisomerase II. *BioEssays*, **13**, 269–275.

POMMIER Y. (1993). DNA topoisomerase I and II in cancer chemotherapy: update and perspectives. *Cancer Chemother. Pharmacol.*, **32**, 103–108.

POTMESIL M. HSIANG Y-H. LIU LF. BANK B. GROSSBERG H. KIRSCHENBAUM S. FORLENZAR TJ. PENZINER A. KANGANIS D. KNOWLES D. TRAGANOS F AND SILBER R. (1988). Resistance of human leukemic and normal lymphocytes to drug-induced DNA cleavage and low levels of DNA topoisomerse II. *Cancer Res.*, **48**, 3537–3543.

PROSPERI E. NEGRI C. MARCHESE G AND ASTALDI RICOTTI GCB. (1994). Expression of the 170 kDa and 180 kDa isoforms of DNA topoisomerase II in resting and proliferating human lymphocytes. *Cell Prolif.*, **27**, 257–267.

ROBERGE M AND GASSER SM. (1992). DNA loops: structural and functional properties of scaffold-attached regions. *Mol. Microbiol.*, **6**, 419–423.

SMITH K. HOULBROOK S. GREENALL M. CARMICHAEL J AND HARRIS AL. (1993). Topoisomerase IIa co-amplification with erbB-2 in human primary breast cancer and breast cancer cell lines–relationship to mAMSA and mitoxantrone sensitivity. *Oncogene*, **8**, 933–938.

SMITH PJ. (1990). DNA topoisomerase dysfunction: a new goal for antitumour chemotheraphy. *BioEssays*, **12**, 167–172.

SMITH PJ AND MAKINSON TA. (1989). Cellular consequences of overproduction of DNA topoisomerase II in an ataxia-telangiectasia cell line. *Cancer Res.*, **49**, 1118–1124.

TUCCARI G. RIZZO A. GIUFFRE G AND BARRESI G. (1993). Immunocytochemical detection of DNA topoisomerase type II in primary breast carcinomas: correlation with clinico-pathological features. *Virchows Arch. A. Pathol. Anat. Histopathol.*, **423**, 51–55.

VERHEIJEN R. KUIJPERS HJH. VAN DRIEL R., BECK JLM. VAN DIERENDONCK JH. BRAKENHOFF GJ AND RAMAEMERS FCS. (1989). Ki-67 detects a nuclear matrix-associated proliferation-related antigen II. Localisation in mitotic cells and association with chromosomes. *J. Cell Sci.*, **92**, 531–540.

WANG JC. (1985). DNA topoisomerases. *Annu. Rev. Biochem.*, **54**, 665–697.

WATT P AND HICKSON ID. (1994). Structure and function of type II DNA topoisomerases. *Biochem. J.*, **303**, 681–695.

WEBB CD. LATHAM MD. LOCK RB AND SULLIVAN DM. (1991). Attenuated topoisomerase II content directly correlates with a low level of drug resistance in a Chinese hamster ovary cell line. *Cancer Res.*, **51**, 6543–6549.

WELLS NJ. ADDISON CM. FRY AM. GANAPATHI R AND HICKSON ID. (1994). Serine-1524 is a major site of phosphorylation ion human topoisomerase II$\alpha$ protein *in vivo* and is a substrate for casein kinase II *in vitro*. *J. Biol. Chem.*, **269**, 29746–29751.

WOESSNER RD. CHUNG TDY. HOFMANN GA. MATTERN MR. MIRABELLI MR. DRAKE FH AND JOHNSON RK. (1990). Differences between normal and *ras*-transformed NIH-3T3 cells in expression of the 170 kD and 180 kD forms of topoisomerase II. *Cancer Res.*, **59**, 2901–2908.

WOESSNER RD. MATTERN MR. MIRABELLI CK. JOHNSON RK AND DRAKE FH. (1991). Proliferation- and cell cycle-dependent differences in expression of the 170 kDa and 180 kDa forms of topoisomerase II in NIH-3T3 cells. *Cell Growth Different.*, **2**, 209–214.

ZWELLING LA. HINDS M. CHAN D. ALTSCHULER E. MAYES J AND ZIPF TF. (1990). Phorbol ester effects on topoisomerase II activity and gene expression in HL-60 human leukemia cells with different proclivities towards monocytoid differentiation. *Cancer Res.*, **50**, 7116–7122.